# When the Offending Catheter Becomes the Rescue Conduit: In Situ Alteplase for Glenn Thrombosis

**DOI:** 10.1016/j.jaccas.2026.109774

**Published:** 2026-08-26

**Authors:** Dragan Nikolić

**Affiliations:** a Clinic for Vascular and Endovascular Surgery, University Clinical Centre of Vojvodina, Novi Sad, Serbia; b Faculty of Medicine, University of Novi Sad, Novi Sad, Serbia

**Keywords:** congenital heart defect, pediatric surgery, thrombosis

**Figure:**
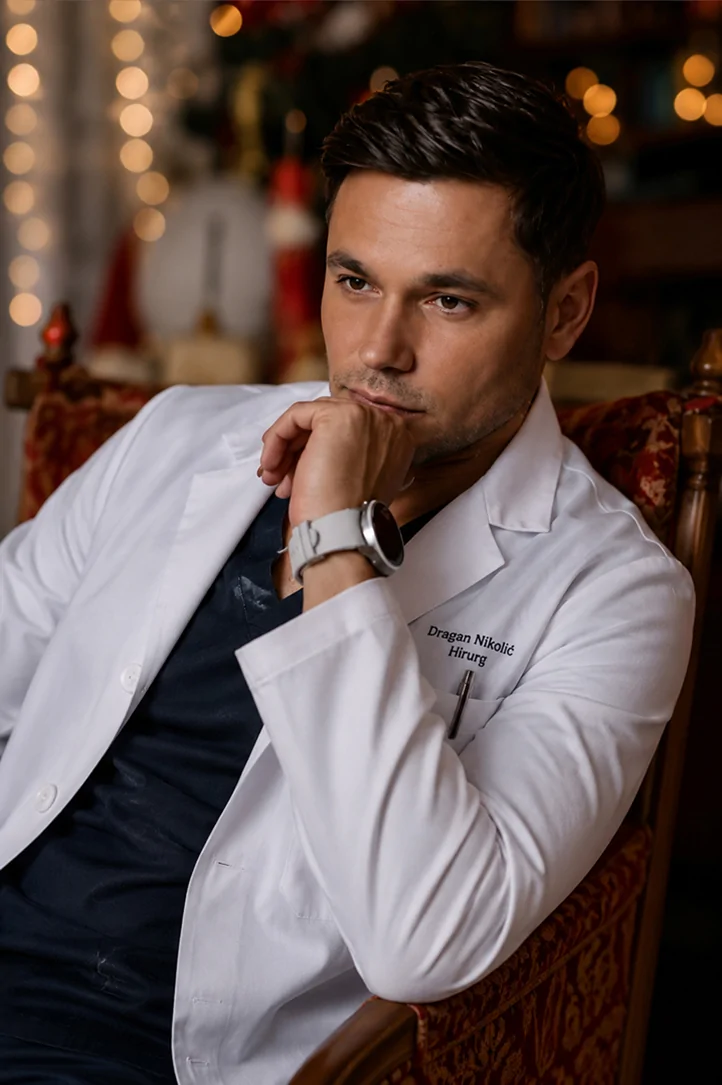

Few hemodynamic catastrophes in pediatric cardiac surgery are as unforgiving as acute obstruction of the cavopulmonary pathway. After a bidirectional Glenn anastomosis, pulmonary blood flow becomes entirely passive, driven by the narrow gradient between the superior vena cava (SVC) and the pulmonary arteries; there is no subpulmonary ventricle to compensate and no reserve to recruit. The therapeutic objective in this physiology is therefore easily misstated. Beyond removing a clot, the true goal is rapid restoration of unobstructed passive cavopulmonary flow, because oxygen delivery in the Glenn circulation depends far more on unimpeded venous return than on ventricular performance. This is also why Glenn-axis thrombosis is a time-dependent emergency rather than a subacute problem: Every hour of persistent obstruction compounds cerebral and upper-body venous hypertension, starves the pulmonary bed of perfusion, and threatens the entire staged single-ventricle pathway. The case reported by Ploteau and et al^1^ in this issue of *JACC: Case Reports* captures that physiology in stark numbers—an SVC pressure of 18 mm Hg, an arterial saturation of 62% on 40% oxygen, and a lactate of 4.2 mmol/L in a 5.8-kg infant—a child at the edge of cavopulmonary circuit collapse.

What makes the report instructive is not the diagnosis but the corner into which it places the clinician. Every conventional escape route was closed. Systemic thrombolysis was untenable only days after mediastinal hemorrhage, surgical re-exploration, and venoarterial extracorporeal membrane oxygenation. Mechanical thrombectomy threatened to shower an already-compromised pulmonary bed with fresh emboli. Surgical thrombectomy in a freshly reopened mediastinum was prohibitive. And the patient had progressed to overt SVC syndrome despite therapeutic heparin, so anticoagulation alone had already declared its insufficiency. This therapeutic impasse gives the report its value: a situation in which standard options were either unavailable or carried risks comparable to inaction.

The authors' solution is disarmingly simple, and its elegance lies in that simplicity. A nontunneled 4-Fr catheter, placed on the first postoperative day, happened to have its tip embedded within the proximal thrombus at the SVC-Glenn junction. Rather than seeking new and hazardous access, the team used the line already in place to infuse alteplase directly into the clot at 0.2 mg/kg/h for 72 hours, holding heparin and resuming anticoagulation in graded fashion once coagulation parameters recovered. The geometry of delivery rewards attention: because the infusion entered the obstructed segment and then followed the re-establishing flow into the pulmonary arteries, a single catheter addressed both the axial SVC thrombus and the distal embolic showering downstream of it. The outcome—complete recanalization, with SVC pressure falling to 9 mm Hg, saturation rising to 84%, and lactate normalizing, without a single bleeding event—was achieved under serial monitoring and prespecified stopping rules, not by good fortune alone.

Read prescriptively, the report risks being reduced to “thrombus present, give alteplase.” Its more useful reading is as a sequence of questions. Is the patient operable? Is systemic thrombolysis acceptable given recent surgery, hemorrhage, or extracorporeal support? Can the thrombus actually be reached? And, decisively here, does an indwelling catheter already cross the lesion? Only when operation and systemic lysis are excluded, and an existing line traverses the clot, does catheter-directed alteplase move from improvisation to a defensible, reproducible choice. Framed this way, the case offers not a reflex but an algorithm.

This experience does not arise in a vacuum. Catheter-directed thrombolysis in single-ventricle physiology has been described before. Hubara et al^2^ reported it in a child with Glenn thrombosis, and contemporary pediatric guidance, including the 2024 American Society of Hematology venous thromboembolism guidelines^3^ and a recent systematic review of thrombolysis in children,^4^ supports site-directed approaches where feasible, while underscoring how thin the underlying evidence remains. The present report extends that literature into a maximally bleeding-prone host, showing that a full 72-hour course can be completed without hemorrhage in precisely the patient most clinicians would consider off-limits.

These strengths sit alongside the limits of the format, which the authors rightly do not overstate. This is a single patient, and one success cannot define a risk-benefit ratio. The strategy was available only because of anatomic serendipity—a catheter tip lying, by chance, within the thrombus—and is therefore opportunistic rather than a plan that can be summoned at will. The denominator, including the failures and complications that single reports seldom capture, remains unknown.

The case also illustrates a principle increasingly visible across cardiovascular medicine: When anatomy and physiology forbid conventional treatment, innovation more often comes from repurposing existing tools than from introducing new ones. The indwelling catheter—ordinarily a leading cause of thrombosis in these fragile children—becomes, when its tip lies within the clot, the very instrument of recanalization, converting an inaccessible lesion into a locally treatable one without new access and without committing the patient to systemic fibrinolysis.

The report is best read as a prompt rather than a conclusion. Observations of this kind become knowledge only when pooled, and a multicenter registry of catheter-directed thrombolysis in single-ventricle patients could begin to define candidate selection, dose, duration, and the complication rates that no single case can reveal. Prevention, ultimately, matters more than rescue: meticulous catheter stewardship, vigilance for early cavopulmonary obstruction, and better defined thromboprophylaxis in the high-risk early postoperative window will always outweigh the most elegant salvage.

Innovation in cardiovascular medicine rarely begins with new technology. More often, it begins with seeing familiar technology through a different physiological lens. Ploteau and colleagues^1^ do not claim to have established a standard, but in a child for whom every conventional rescue was contraindicated, they show that the catheter responsible for the thrombosis could become the instrument that restored the circulation—a reframing, and a contribution, worth having.

## Funding Support and Author Disclosures

The author has reported no relationships relevant to the contents of this paper to disclose.
